# *Fructus mume* extracts alleviate cognitive impairments in 5XFAD transgenic mice

**DOI:** 10.1186/s12906-016-1033-0

**Published:** 2016-02-06

**Authors:** Jung-Cheol Park, Jinhua Ma, Won Kyung Jeon, Jung-Soo Han

**Affiliations:** 1 Department of Biological Sciences, Konkuk University, 1 Hwayang-dong, Gwangjin-gu, Seoul 143-701 Republic of Korea; 2 Herbal Medicine Research Division, Korea Institute of Oriental Medicine, Daejeon, 305-811 Republic of Korea

**Keywords:** *Fructus mume*, Alzheimer’s disease, Cognition, Choline acetyltransferase, 5XFAD

## Abstract

**Background:**

*Fructus mume (F. mume)* has been used as a traditional treatment for ulcer, cough, and digestive problems for many years in Asian countries. Previous studies have demonstrated that *F. mume* extracts alleviate cognitive deficits in rats with chronic cerebral hypoperfusion and in mice with scopolamine treatments. The present experiment was conducted to examine the effects of *F. mume* on cognitive impairments in 5XFAD transgenic mice with five familial Alzheimer’s disease (AD) mutations.

**Methods:**

*F. mume* was administered daily to 5XFAD mice at 12 weeks of age and continued for 90 days. Cognitive function was evaluated using a spatial memory version of the Morris water maze task, the object/location novelty recognition test, and contextual fear conditioning at 24 weeks of age. To elucidate the possible mechanisms underlying the memory improving effects of *F. mume* in 5XFAD mice, we examined alterations in hippocampal cholinergic function.

**Results:**

Vehicle-treated 5XFAD mice exhibited hippocampus-dependent memory impairments compared with non-transgenic littermates, which was reversed in *F. mume*-treated 5XFAD mice. In addition, reduced hippocampal choline acetyltransferase (ChAT) levels in 5XFAD mice were reversed by *F. mume* treatment, indicating that *F. mume* enhances the effects of cholinergic neuronal function.

**Conclusions:**

*F. mume* may have therapeutic effects on cognitive impairments in AD.

## Background

*Fructus mume (F. mume),* the processed unripe fruit of *Prunus mume*, has been used as a traditional treatment for many years in Asian countries. *F. mume* has been reported to be effective in a rat model of colitis induced by 2,4,6-trinitrobenzene sulfonic acid [1], and is inhibitory to pro-inflammatory mediators in lipopolysaccharide (LPS)-stimulated RAW 264.7 cells [2]. We recently reported that *F. mume* treatment can alleviate impaired cognitive function in a rat model of vascular dementia (VaD) induced by a bilateral common carotid artery occlusion (BCCAo) [3–6]. We also examined the effects of *F. mume* on scopolamine-induced cognitive impairment in mice [7]. In agreement with the results of the BCCAo study, treatment with *F. mume* improved cognitive impairments in scopolamine-injected mice [7].

These latter two studies [6, 7] also found that *F. mume* has anti-inflammatory effects and enhances central cholinergic function. Based on this reported action of *F. mume,* the present experiment was conducted to examine the therapeutic potential of *F. mume* for Alzheimer’s disease (AD)*,* using an animal AD model.

AD is characterized by a progressive loss of cognitive function. The neuropathological hallmarks are the accumulation of extracellular senile plaques and intraneuronal fibrillary tangles, as well as the loss of basal forebrain cholinergic neurons [8, 9]. In particular, numerous studies indicate that basal forebrain cholinergic neurons, which provide the major innervation of the hippocampus and cortex, play an important role in cognitive function [10–12]. The loss of basal forebrain cholinergic neurons has been reported in the brains of patients with VaD and AD. In addition, neurodegeneration of basal forebrain cholinergic neurons has been observed in the brains of BCCAo rats and AD transgenic mice [13–17]. Therefore, given that *F. mume* ameliorated BCCAo-induced and scopolamine-induced cognitive impairments, we expected beneficial effects of *F. mume* on cognitive deficits in AD.

To investigate whether *F. mume* mitigates cognitive impairments in AD, we used 5XFAD transgenic mice co-overexpressing human amyloid precursor protein (APP) and presenilin-1 (PS1) with five familial AD (FAD) mutations. 5XFAD mice generate and accumulate cerebral amyloid-β (Aβ) 42 and begin to develop visible amyloid deposits at 2 months of age [18]. 5XFAD mice exhibit memory decline, especially in hippocampus-dependent behavioral tasks, at 4–6 months of age [19, 20]. In addition, 5XFAD mice exhibit reduced choline acetyltransferase (ChAT) levels in the hippocampus and loss of cholinergic neurons in the medial septum (MS) and the vertical limb of diagonal band (VDB) at 6 months of age [21]. In the present study, 5XFAD mice were administered *F. mume* for approximately 3 months and then followed several behavioral tasks for assessing cognitive function. *F. mume* treatment alleviated cognitive impairments via increases in hippocampal ChAT levels.

## Methods

### Preparation of *F. mume* extracts

*F. mume* was purchased from a commercial supplier (Kwangmyung-Dang, Ulsan, Korea) in 2010. It was identified by the Herbal Quality Control Team and deposited at the Creative Research Laboratory, KIOM (Korea). Dried *F. mume* was pulverized and extracted with distilled water (2 kg/8 L) for 2 h below 100 °C in an ultrasound-assisted extractor (OM30-EP; Sonimedi, Korea). All extracts were concentrated under vacuum using a rotary evaporator after filtration and were then dried for 48 h at 40 °C by using an extract vacuum drier (Exdryer, Sonimedi, Korea) to yield a powder extract (324.5 g, 16.225 % yield). The powder extract was suspended in sterilized distilled water at the appropriate concentrations. An HPLC assay was performed with citric acid as a standard maker for quality control of the *F. mume* extract composition in each experiment. HPLC was performed using two Waters 515 pumps, a 2996 photodiode array detector, and a Phenomenex Synergi Hydro RP-80A (4 m, 4.6 x 250 mm i.d.). The mobile phase was composed of acetonitrile (A) and 0.1 % phosphoric acid (B) with a linear gradient elution: 0 min, 100 % B; 15 min, 3.8 % A. *F. mume* extract was filtered on membrane filters with a 0.45 mm pore size (Millipore) and a 10 L injection volume. Citric acid was detected at a wavelength of 220 nm. The crude extract was analyzed in triplicate, and the citric acid content was found to be about 18.68 % [6, 22].

### Animals and *F. mume* extract administration

The generation of 5XFAD mice (Tg6799 line) has been described previously [18]. These mice co-express and co-inherit both mutant human APP (695) with the Swedish (K670N, M671L), Florida (I716V), and London (V717I; FAD) mutations and human PS1 harboring two FAD mutations, M146L and L286V. The expression of these transgenes is regulated by neuron-specific mouse Thy-1 promoters to drive overexpression in the neurons. The 5XFAD strain (B6/SJL genetic background) was maintained by crossing hemizygous transgenic mice with non-transgenic B6/SJL breeders, and 5XFAD transgenic mice were used for the experiments with non-transgenic littermates as controls. Before the experiments, genomic DNA was extracted from the tail tips of all mice and genotyping was performed by polymerase chain reaction analysis. 23 non-transgenic controls (18 male, 5 female) and 38 5XFAD (28 male, 10 female) mice were used for the experiments. All mice had access to food and water ad libitum and were housed under a 12–12 h light–dark cycle at 22–24 °C. All behavioral experiments were performed during the light phase.

Preparation of *F. mume* has been described previously [6]. Mice were administered *F. mume* extract (200 mg/kg) or distilled water as a vehicle control by oral gavage. Treatment began when the mice were 12 weeks of age and continued until they were sacrificed, for approximately 3 months. The Institutional Animal Care and Use Committee of Konkuk University approved all protocols described in this report.

### Morris water maze task

The Morris water maze test pool was 1.83 m in diameter, and 0.58 m in height, with a 0.2 m in diameter hidden platform and was surrounded by a white curtain marked with extra-visual cues. The pool was filled with 24–27 °C opaque water. Mice were trained on four trials per day for 8 consecutive days. Mice were allowed to search for the platform for 60 s; if they did not find the platform, they were placed on the platform manually, where they were held for 15 s. After the trials were completed, the mice were dried and placed back into their home cages. To assess memory retention, a probe test was performed on training day 5, 7, and 9. During the probe test, platform was removed and mice were allowed to swim for 30 s. In all trials, the movements of the mice were recorded and analyzed using a HVS image tracking system (Hampton, UK).

### Contextual fear conditioning task

Contextual fear conditioning task was conducted in square (18 cm W x 18 cm D x 30 cm H) chambers equipped with a steel-grid floor through which foot-shock could be delivered (Coulbourn, PA, USA). The task consisted of habituation, training, and test sessions. During habituation, the mice were placed in a conditioning chamber and allowed to explore without a foot shock for 12 min. On the next day, during training, mice were placed in the conditioning chamber for 148 s, and then received three unsignaled foot shocks (0.75 mA, 2 s) at 30-s intervals. The test session was scheduled 24 h after training. During the test, mice were placed into the same conditioning chamber, and contextual fear memory was evaluated by scoring freezing behavior for 3 min by an investigator blind to condition. In addition, the baseline freezing behavior was analyzed during the 2 min before the delivery of the foot shock during the training session.

### Object/location novelty recognition test

The object/location novelty recognition test consisted of four sessions: habituation, sample object phase, novel object phase, and novel object location phase. Each mouse was individually habituated to the open field box (50 × 50 cm) for 15 min. On the next day during the sample object phase, two identical sample objects were placed in the two corners of the box, and the mice were allowed to freely explore the arena and objects for 10 min. The novel object phase was scheduled 24 h later, in which the mice were placed into the arena again, and one of the sample objects was replaced with a novel object. Mice were allowed to explore the arena for 5 min, and the time spent exploring each object was scored for 2 min. 24 h later, during the novel object location phase, one of the two objects that were used in novel object phase was moved to a novel place in the arena, and the mice were tested in the same way as before. The discrimination ratio was calculated as the ratio of time spent exploring the novel object or the object moved to novel location to the total time spent exploring the two objects.

### Western blot analysis

One week after the behavioral experiments, 9 mice per group were decapitated and the hippocampal or cortical samples were dissected and snap-frozen. For the total protein extracts, individual tissue samples were homogenized in ice-cold buffer containing 20 mM Tris (pH 7.5), 5 % glycerol, 1.5 mM EDTA, 40 mM KCl, 0.5 mM dithiothreitol, and protease inhibitors. The homogenates were then centrifuged at 14,000 rpm for 1 h at 4 °C, and the supernatant was harvested and stored at−80 °C. The concentration of the protein extracts was determined by the Bradford assay. The protein extracts were separated by SDS-PAGE and transferred to a PVDF membrane using the Mini Trans-Blot Cell (Bio-Rad). After blocking, the membranes were incubated with anti-ChAT antibody (goat polyclonal; millipore, 1:1000), anti-β-actin antibody (sigma, 1:5000), and then with HRP-conjugated secondary antibodies. The signals were visualized by an ECL system and developed onto hyperfilm. The relative ChAT expression level was determined by densitometry using the Image Gauge software (Fujifilm) and was normalized to β-actin levels.

### Immunohistochemistry

One week after the behavioral experiments, 6 mice per group were euthanized by ketamine and xylazine and were intracardially perfused with 4 % paraformaldehyde (PFA) in phosphate buffered saline (PBS). Following fixation, the brains were removed to 4 % PFA and 30 % sucrose solutions, respectively, in order. Free-floating brain sections (40 μm) were obtained from frozen-section mice brains using microtome (Leica). Endogenous peroxidase activity in the brain sections was quenched by incubation in 3 % H_2_O_2_/10 % MeOH in PBS. The sections were then incubated for 1 h at room temperature in PBS with 0.3 % Triton-X 100 containing 10 % fetal horse serum (GIBCO). The sections were incubated with anti-ChAT antibody (goat polyclonal; Millipore, 1:1000) overnight and then incubated for 1 h with the appropriate biotinylated secondary antibodies (Vector, 1:500) and for 2 h in extravidin peroxidase conjugate (Sigma Aldrich, 1:1000). Finally, the sections were reacted with a 3,3′-diaminobenzidine (DAB) substrate kit (Vector) and mounted onto resin coated slides. After dehydration in a series of ethanol and clearance in xylene, the slides were covered with a coverslip using permount reagent. To quantify the number of ChAT-positive cells, 8 sections from the medial septum region (between +1.2 and +0.8 mm anterior to the bregma) and 4 sections from the substantia innominata region (between +0.3 and +0.1 mm anterior to the bregma) were selected from each individual. To capture the images, light microscopy was conducted on ECLIPSE Ni-U microscope (Nikon, Japan) equipped with a ProgRes CFscan camera (JENOPTIK, Germany). After the same threshold level and region of interest were applied to each image, the number of ChAT-positive cells was counted using MetaVue software.

### Statistical analysis

One-way analysis of variance (ANOVA) or two-way ANOVA with repeated measurements was conducted to assess the effects of the *F. mume* extract on the changes in hippocampal and cortical ChAT levels and the cognitive impairments in 5XFAD mice. Post-hoc analyses (Least Significant Difference test) were subsequently performed to determine the effects of *the F. mume* treatment. *P* values of less than 0.05 were considered significant, unless otherwise specified. The data were expressed as the mean ± standard error of the mean (SEM).

## Results

### F. mume alleviated cognitive impairments in 5XFAD mice

**Fig. 1:**
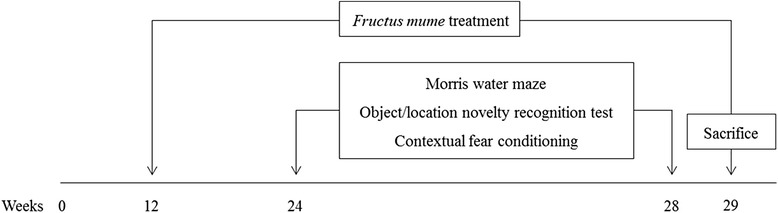
Experimental procedure. Daily treatment with *F. mume* (200 mg/kg) or vehicle was initiated at 12 weeks of age and continued for 90 days. Behavioral experiments including the Morris water maze, the object/location novelty recognition test, and contextual fear conditioning were sequentially conducted at 24 weeks of age

**Fig. 2:**
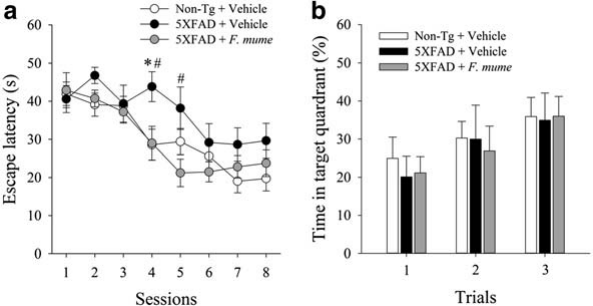
Treatment with *F. mume* ameliorated impairments in spatial learning on the Morris water maze in 5XFAD mice. **a** During the hidden platform training sessions, vehicle-treated 5XFAD mice exhibited a significant learning deficit during the spatial learning training compared with vehicle-treated non-transgenic mice (*p < 0.05). *F. mume* treatment alleviated this impairment in spatial learning (^#^ *p* < 0.05 versus vehicle-treated 5XFAD mice) on sessions 4 and 5. **b** Probe trials were conducted before the acquisition trials on sessions 5, 7, and 9 to assess the retention of spatial memory. The percentage of time spent in the target quadrant was analyzed over 30 s. No significant differences were observed between the groups (*n* = 8 – 14 per group). All the data are presented as the mean ± SEM

To examine the effect of *F. mume* on cognitive impairments in 5XFAD mice, several hippocampus-dependent behavioral tasks were conducted (Fig. 1). We first conducted the Morris water maze task to assess spatial memory. It has previously been reported that 5XFAD mice exhibit memory impairment in the Morris water maze task at 6 months of age [23]. In the present experiment, all mice improved in locating the submerged platform across the 8 training sessions, as shown by decreasing latency (Fig. 2a; two-way ANOVA with 4 trials/session as a repeated measure; main effect of training: *F*_*(*7,203)_ = 20.01, *p* < 0.001). Neither the main effect of treatment (*F*_*(*2,29)_ = 1.97, *p* = 0.16) nor the interaction effects of treatment x training (*F*_*(*14,203)_ = 1.51, *p* = 0.11) were significant. Post-hoc analyses revealed that performance of 5XFAD mice was poorer than those of vehicle-treated control at the session 4 (*p* < 0.05) and that performance of *F. mume*-treated 5XFAD mice was improved compared with vehicle-treated 5XFAD mice at the session 4 and 5 (*p* < 0.05) (Fig. 2a). However, no significant difference between the groups was found during the probe trials for the assessment of spatial memory retention (Fig. 2b).

**Fig. 3:**
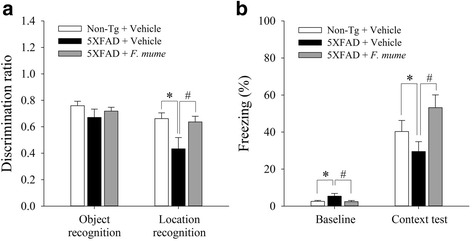
*F. mume* treatment ameliorated impairments in recognition and fear memory in 5XFAD mice. **a** The object/location novelty recognition test consisted of a novel object and novel location phase. During the object phase, no significant differences in the preference for a novel object were observed between the groups, whereas, during the subsequent novel location phase, vehicle-treated 5XFAD mice exhibited less preference for the object moved to the novel location than vehicle-treated non-transgenic mice (*p < 0.05), which was reversed by *F. mume* treatment (^#^ *p* < 0.05). **b** During contextual fear conditioning, mice were exposed to a context (conditioning chamber) for 148 s and then received three unsignaled foot shocks (0.75 mA, 2 s, 30 s interval). Context-associated fear memory was measured by observing freezing behavior 24 h later. During the test phase, vehicle-treated 5XFAD mice exhibited less freezing behavior than vehicle-treated non-transgenic mice (*p < 0.05), which was reversed by treatment of *F. mume* (^#^ *p* < 0.05). During the baseline, prior to the foot shock delivery, vehicle-treated 5XFAD mice exhibited more activity than mice in the other groups (*^,#^ *p* < 0.05) (*n* = 16 – 17 per group). All the data are presented as the mean ± SEM

Mice were then tested on object recognition task and the location novelty recognition task. Briefly, mice were initially exposed to two identical objects and re-exposed to the objects 24 h later when one of the objects was replaced with a novel object. After this novel object phase, the mice were tested again in a location novelty recognition test in which one of the objects was moved to a novel location in the arena. The location novelty recognition task is known to be sensitive to hippocampal damage [24]. In the novel object phase, no significant difference in the discrimination ratio was observed between the groups. However, in the new location phase, a difference in the discrimination ratio between groups was found. One-way ANOVA revealed significant between-group effects (F_(2, 46)_ = 4.360, *p* < 0.05). Post-hoc analyses revealed that vehicle-treated 5XFAD mice spent less time exploring the object moved to novel location compared with the vehicle-treated control mice (Fig. 3a) and that the discrimination ratio in *F. mume*-treated 5XFAD mice was increased relative to vehicle-treated 5XFAD mice (*p* < 0.05) (Fig. 3a).

The final task was contextual fear conditioning, in which mice learned an association between a context (a conditioning chamber) and an aversive stimulus (a foot shock). An earlier study reported that fear memory was impaired in 5XFAD mice [19]. We measured basal levels of freezing behavior in the conditioning chamber before the foot shock delivery. Even though the basal levels of freezing behavior were low in all the groups, one-way ANOVA revealed significant between-group effects (F_(2, 46)_ = 3.08, *p* < 0.05). Specifically, vehicle-treated 5XFAD mice exhibited slightly more freezing behavior than vehicle-treated non-transgenic mice. In contrast, the basal levels of freezing behavior were decreased in *F. mume*-treated 5XFAD mice compared with vehicle-treated 5XFAD mice. During the test session conducted 24 h after receiving the foot shock, the freezing levels were increased in all the groups, but with significant between-group differences (F_(2, 45)_ = 7.84, *p* < 0.01). Post-hoc analyses revealed that vehicle-treated 5XFAD mice exhibited reduced freezing behavior compared with vehicle-treated non-transgenic mice (*p* < 0.05), which implies that the fear memory was impaired in 5XFAD mice. This fear memory impairment was rescued by *F. mume* treatment (*p* < 0.05) (Fig. 3b). Together these results suggest that *F. mume* is effective in improving cognitive dysfunction in 5XFAD mice.

### F. mume increased levels of choline acetyltransferase expression in the hippocampus of 5XFAD mice

**Fig. 4:**
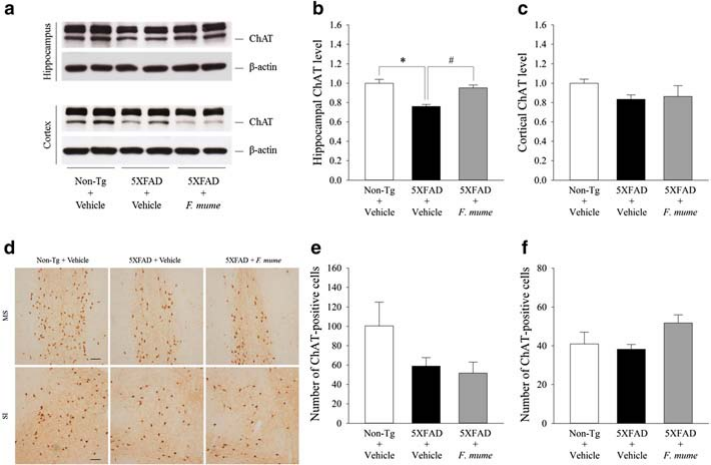
*F. mume* treatment increased hippocampal ChAT expression in 5XFAD mice. **a** Hippocampal or cortical ChAT expression level was analyzed by western blot (normalized to non-transgenic + vehicle). **b** The expression levels of hippocampal ChAT were significantly reduced in vehicle-treated 5XFAD mice compared with vehicle-treated non-transgenic mice (*p < 0.05). *F. mume* treatment mitigated this reduction (^#^ *p* < 0.05) (*n* = 6 per group). **c** No significant differences in expression levels of cortical ChAT were found between the groups (*n* = 8 – 9 per group). **d** Representative ChAT-positive neurons in the medial septum (MS) and substantia innominata (SI) are shown. Scale bar = 100 μm. **e** The number of ChAT-positive neurons in the MS and vertical limb of the diagonal band (VDB), which provide cholinergic innervation to the hippocampus, was counted (*n* = 6 per group). **f** The number of ChAT-positive neurons in the SI and horizontal limb of the diagonal band (HDB), which provide cholinergic innervation to the cortex, was counted (*n* = 4 per group). No significant differences were observed in the number of ChAT-positive neurons in the basal forebrain between the groups. All the data are presented as the mean ± SEM

It has been reported that the septo-hippocampal cholinergic system is impaired in 5XFAD mice [21, 25]. To elucidate the possible mechanisms contributing to the memory improving effect of *F. mume*, hippocampal and cortical ChAT expression levels were measured by western blot analysis (Fig. 4a). One-way ANOVA revealed significant between-group effects on the hippocampal ChAT levels (F_(2, 15)_ = 16.435, *p* < 0.01) (Fig. 4a and b). Post-hoc analyses revealed that hippocampal ChAT levels was significantly reduced in vehicle-treated 5XFAD mice compared with vehicle-treated non-transgenic mice, which was reversed by *F. mume* treatment (*p* < 0.05) (Fig. 4a and b). No significant between-group differences were observed in cortical ChAT expression levels (Fig. 4a and c).

To further examine the status of cholinergic neurons in the basal forebrain of these mice, the number of ChAT-positive neurons in the medial septum (MS) and the vertical limb of the diagonal band (VBD), which provide the main cholinergic innervation to the hippocampus, were counted using immunohistochemistry (Fig. 4d). Consistent with previous studies [19], the number of ChAT-positive neurons was reduced in the vehicle-treated 5XFAD mice and *F. mume*-treated 5XFAD mice (Fig. 4d and e). Furthermore, the number of ChAT-positive neurons that project to the cortex within the horizontal limb of the diagonal band (HDB), the magnocellular preoptic nucleus (MCPO), and the substantia innominata (SI) was not significantly different between groups (Fig. 4d and f). These results indicate that *F. mume* ameliorates impaired cholinergic function via the up-regulation of hippocampal ChAT levels rather than by prevention of cholinergic neurodegeneration in 5XFAD mice.

## Discussion

*F. mume*, the smoked fruit of *Prunus mume* SIEB. *et* Zucc. (Rosaceae family), has been used to treat gastrointestinal disease, ulcer, and cough in Asian countries for thousands of years [1]. The therapeutic effects and mechanisms *F. mume* extract on macrophage-mediated inflammation were examined in LPS-stimulated RAW 264.7 cells. *F. mume* treatment inhibited the LPS-induced production of nitric oxide, prostaglandin E2, and interleukin-6 production. *F. mume* treatment also suppressed the signaling of mitogen-activated protein kinase and nuclear factor-κB activated by LPS stimulation [2]. In addition, *F. mume* extract was an effective treatment in an animal model of colitis [1].

Based on the results reported above, additional studies were conducted to examine the effects of *F. mume* extract on chronic BCCAo-induced microglial activation and the resultant cognitive improvement. *F. mume*-treated BCCAo rats exhibited less inflammation and more improvements in spatial memory compared with vehicle-treated BCCAo rats [6, 26]. However, it is possible that *F. mume* attenuates basal forebrain cholinergic neurons impairments in rats with chronic BCCAo based on the observation of neurodegeneration in the basal forebrain cholinergic neurons of rats with chronic BCCAo [13] as well as observation that *F. mume* rescued scopolamine-induced spatial memory and cholinergic system impairments [7].

Hippocampal ChAT levels are markedly reduced in the brains of AD patients [27]. Studies have demonstrated that treatments leading to the up-regulation of hippocampal acetylcholine (ACh) levels reverse memory deficits [28]. For example, cognitive deficits induced by fimbria-fornix lesions are attenuated by ACh-secreting fibroblasts implanted into the hippocampus [12]. Similarly, hippocampal grafts of fetal neuronal tissue rich in cholinergic neurons reverse memory deficits produced by a variety of manipulations including septal, hippocampal, fimbria-fornix lesions, and selective 192 IgG-saporin-induced lesions of the septo-hippocampal ACh projection [29–31].

In addition, the expression level of acetylcholinesterase (AChE), which breaks down ACh, has been examined in relation to AD pathology. Indeed, an Aβ-induced AChE increase has been observed both vitro and in vivo [32–34]. There was a positive correlation between the Aβ level and AChE activity in human cerebrospinal fluid [35]. AChE may interact with Aβ to accelerate the deposition of amyloid plaque in the brains of AD patients [36]. To counteract this detrimental action of AChE in AD, AChE inhibitors such as donepezil are widely used. The therapeutic effect of AChE inhibitors has also confirmed in a mouse model of AD [37].

Therefore, the present study examined the efficacy of *F. mume* on cognitive deficits in AD using 5XFAD transgenic mice. Specifically, cognitive status was measured in vehicle-treated non-transgenic, vehicle-treated 5XFAD, and *F. mume-*treated 5XFAD mice using the Morris water maze, location novelty recognition, and contextual fear conditioning tasks. Impairments in the spatial version of the Morris water maze task, location novelty recognition task, and contextual fear conditioning task were observed in vehicle-treated 5XFAD mice relative to vehicle-treated non-transgenic mice, which is consistent with earlier reports [20, 38]. These cognitive impairments were alleviated in the *F. mume*-treated 5XFAD mice.

Moreover, we measured hippocampal and cortical ChAT levels using western blot analysis and counted the number of cholinergic neurons in the basal forebrain using immunohistology in the vehicle-treated non-transgenic, vehicle-treated 5XFAD, and *F. mume*-treated 5XFAD mice, to reveal the underlying action mechanism of *F. mume.* A significant difference in the number of ChAT-positive neurons in the MS and the VBD, which innervate to the hippocampus, and the hippocampal ChAT levels was observed between vehicle-treated non-transgenic and vehicle-treated 5XFAD mice, in agreement with previous reports [21, 25]. Hippocampal ChAT levels were restored in the *F. mume*-treated 5XFAD mice. However, no statistical difference between vehicle-treated non-transgenic and vehicle-treated 5XFAD mice was observed in the number of cortically projecting ChAT-positive neurons in the HDB, MCPO, and SI.

*F. mume*-treatment is reported to increase ChAT levels and decrease AChE levels in the hippocampus [7]. A study that explores AChE levels in the hippocampus and cortex of *F. mume*-treated 5XFAD mice is needed to support the hypothesis that *F. mume* acts on cholinergic neurons. In addition to improving cholinergic function, the anti-inflammatory effects of *F. mume* have been reported in a rat model of VaD using chronic cerebral hypoperfusion [6]. In that study, the expression levels of neuroinflammation markers such as activated microglia cells were reduced by *F. mume*. However, several studies from those in transgenic mice (including 5XFAD mice) to those in AD patients have demonstrated the crucial roles of neuroinflammation in AD pathology [18, 39, 40]. Thus, it is worth noting that the anti-inflammatory effect of *F. mume* may also contribute to cognitive deficit improvements.

## Conclusion

The present study demonstrated that *F. mume* improved cognitive impairments in 5XFAD mice. These results are consistent with previous reports in a chronic cerebral hypoperfusion rat model of VaD and in a scopolamine-induced model of memory impairment in mouse. *F. mume* also increased hippocampal ChAT expression levels, which provides evidence that *F. mume* has a therapeutic effect on impaired cholinergic functions in AD pathology. Therefore, these results suggest that *F. mume* may be a novel agent for the cognitive improvement and the restoration of cholinergic systems in AD.

## Abbreviations

ACh: acetylcholine
AChE: acetylcholinesterase
AD: Alzheimer’s disease
APP: amyloid precursor protein
Aβ: amyloid beta
ChAT: choline acetyltransfersase
*F. mume*: *Fructus* mume
FAD: familial AD
MS: medial septum
PS1: presenilin-1
SI: substantia innominata
VDB: vertical diagonal band
