# Erratum to: *Fructus mume* extracts alleviate cognitive impairments in 5XFAD transgenic mice

**DOI:** 10.1186/s12906-016-1414-4

**Published:** 2016-10-31

**Authors:** Jung-Cheol Park, Jinhua Ma, Won Kyung Jeon, Jung-Soo Han

**Affiliations:** 1 Department of Biological Sciences, Konkuk University, 1 Hwayang-dong, Gwangjin-gu, Seoul, 143-701 Republic of Korea; 2 Herbal Medicine Research Division, Korea Institute of Oriental Medicine, Daejeon, 305-811 Republic of Korea

## Erratum

Following publication of the original article [1] it was brought to our attention that a grant number had been missed out in the acknowledgements of this article. At current, the acknowledgements section reads as follows:

We would like to acknowledge the financial support from the R&D Convergence Program of NST (National Research Council of Science & Technology, G15120 and CRC-15-04-KIST) of Republic of Korea.

However, the revised version should read as:

We would like to acknowledge the financial support from the R&D Convergence Program of NST (National Research Council of Science & Technology, G15120 and CRC-15-04-KIST) of Republic of Korea and a grant (K15311) from the KIOM.
